# TUBB3 Arg262His causes a recognizable syndrome including CFEOM3, facial palsy, joint contractures, and early-onset peripheral neuropathy

**DOI:** 10.1007/s00439-021-02379-9

**Published:** 2021-10-15

**Authors:** Mary C. Whitman, Brenda J. Barry, Caroline D. Robson, Flavia M. Facio, Carol Van Ryzin, Wai-Man Chan, Tanya J. Lehky, Audrey Thurm, Christopher Zalewski, Kelly A. King, Carmen Brewer, Konstantinia Almpani, Janice S. Lee, Angela Delaney, Edmond J. FitzGibbon, Paul R. Lee, Camilo Toro, Scott M. Paul, Omar A. Abdul-Rahman, Bryn D. Webb, Ethylin Wang Jabs, Hans Ulrik Moller, Dorte Ancher Larsen, Jayne H. Antony, Christopher Troedson, Alan Ma, Glad Ragnhild, Katrine V. Wirgenes, Emma Tham, Malin Kvarnung, Timothy James Maarup, Sarah MacKinnon, David G. Hunter, Francis S. Collins, Irini Manoli, Elizabeth C. Engle

**Affiliations:** 1 Department of Ophthalmology, Boston Children’s Hospital, Boston, Massachusetts 02115, USA; 2 Department of Ophthalmology, Harvard Medical School, Boston, Massachusetts 02115, USA; 3 Department of Neurology, Boston Children’s Hospital, Boston, Massachusetts 02115, USA; 4 Howard Hughes Medical Institute, Chevy Chase, MD; 5 Department of Radiology, Boston Children’s Hospital, Boston, Massachusetts 02115, USA; 6 Department of Radiology, Harvard Medical School, Boston, Massachusetts 02115, USA; 7 National Human Genome Research Institute, National Institutes of Health, Bethesda, MD 20892 USA; 8 EMG Section, National Institute of Neurological Disorders and Stroke, NIH, Bethesda, MD 20892-1404 USA; 9 Neurodevelopmental and Behavioral Phenotyping Service, National Institute of Mental Health, Bethesda, MD 20892; 10 Audiology Unit, Otolaryngology Branch, National Institute on Deafness and Other Communication Disorders, National Institutes of Health; 11 Clinical Research Fellow, NIH, 30 Convent Dr., Room 203, Bethesda, MD 20892; 12 Clinical Director, NIDCR & Deputy Director for Intramural Clinical Research, NIH, 10 Center Dr. Room 5-2531, Bethesda, MD 20892-1470; 13 Pediatric Endocrinology and Metabolism Group, National Institute of Child Health and Human Development, NIH, Bethesda, MD, 20892; 14 Laboratory of Sensorimotor Research, National Eye Institute, NIH, Bethesda, MD 20892 USA; 15 Staff Clinician, NIH Undiagnosed Diseases Program. National Human Genome Research Institute, Bethesda, MD 20892; 16 Medical Staff Section, Rehabilitation Medicine Department, NIH Clinical Center, 10 Center Drive, Bethesda, MD 20892-1604; 17 Departments of Biomedical Engineering and Physical Medicine & Rehabilitation, JHU School of Medicine, Baltimore, MD 21205; 18 Chief, Division of Medical Genetics, University of Mississippi Medical Center, 2500 North State Street, Jackson, MS 39216; 19 Department of Genetics and Genomic Sciences, Icahn School of Medicine at Mount Sinai, New York, New York 10029, USA; Present Address: Division of Genetics and Metabolism, Department of Pediatrics, University of Wisconsin - Madison, Madison, WI, USA; 20 Department of Genetics and Genomic Sciences; Department of Cell, Developmental, and Regenerative Biology; Department of Pediatrics; Icahn School of Medicine at Mount Sinai, New York, New York 10029; 21 Aarhus University, Denmark (Emeritus); 22 Aarhus University, Denmark; 23 Children’s Hospital Westmead, NSW Australia; 24 Specialty of Genomic Medicine, University of Sydney, Sydney Australia; 25 Department of Medical Genetics, University Hospital North Norway; 26 Department of Medical Genetics, Oslo University Hospital, Norway; 27 Institute of clinical medicine, University of Oslo; 28 Consultant Clinical Genetics, Karolinska University Hospital, L5:03, 171 76 Stockholm, Sweden; 29 Department of Molecular Medicine and Surgery, Karolinska Institutet, 171 76 Stockholm, Sweden; 30 Department of Clinical Genetics, Karolinska University Hospital, Stockholm, Sweden; 31 Department of Genetics, Kaiser Permanente, Los Angeles, CA, USA; 32 Office of the Director, NIH, Bethesda, MD 20892 USA; 33 Kirby Center, Boston Children’s Hospital, Boston, Massachusetts, 02115; 34 Department of Neurology, Harvard Medical School, Boston, Massachusetts, 02115

**Keywords:** TUBB3, Kallmann Syndrome, CFEOM, facial weakness, arthrogryposis, peripheral neuropathy, tubulinopathy

## Abstract

Microtubules are formed from heterodimers of alpha and beta tubulin, each of which has multiple isoforms encoded by separate genes. Pathogenic missense variants in multiple different tubulin isoforms cause brain malformations. Missense mutations in *TUBB3*, which encodes the neuron-specific beta-tubulin isotype, can cause congenital fibrosis of the extraocular muscles type 3 (CFEOM3) and/or malformations of cortical development, with distinct genotype-phenotype correlations. Here, we report fourteen individuals from thirteen unrelated families, each of whom harbors the identical NM_006086.4(TUBB3):c.785G>A (p.Arg262His) variant resulting in a phenotype we refer to as the TUBB3 R262H syndrome. The affected individuals present at birth with ptosis, ophthalmoplegia, exotropia, facial weakness, facial dysmorphisms, and, in most cases, distal congenital joint contractures, and subsequently develop intellectual disabilities, gait disorders with proximal joint contractures, Kallmann syndrome (hypogonadotropic hypogonadism and anosmia), and a progressive peripheral neuropathy during the first decade of life. Subsets may also have vocal cord paralysis, auditory dysfunction, cyclic vomiting, and/or tachycardia at rest. All fourteen subjects share a recognizable set of brain malformations, including hypoplasia of the corpus callosum and anterior commissure, basal ganglia malformations, absent olfactory bulbs and sulci, and subtle cerebellar malformations. While similar, individuals with the TUBB3 R262H syndrome can be distinguished from individuals with the TUBB3 E410K syndrome by the presence of congenital and acquired joint contractures, an earlier onset peripheral neuropathy, impaired gait, and basal ganglia malformations.

## INTRODUCTION

Microtubules form a cellular scaffold that is essential for proper development and function of the nervous system. They provide structure to the neuron, and kinesin and dynein molecular motor proteins travel along microtubules to transport molecular cargos throughout the cell. Microtubules are composed of heterodimers of alpha- and beta-tubulin, which form into protofilaments. The protofilaments then form into hollow dynamic microtubules, which constantly undergo polymerization (growth) and depolymerization (catastrophe). Each tubulin monomer has many isoforms, some of which have cell-specific expression patterns. Each is also subject to multiple forms of post-translational modification. This variety leads to a “tubulin code” that coordinates the many functions of microtubules in diverse cell types (Janke 2014).

Heterozygous missense variants altering tubulin isotypes cause a variety of neurological phenotypes, ranging from lissencephaly to polymicrogyria to isolated eye movement disorders (Bahi-Buisson et al. 2014; Fallet-Bianco et al. 2014; Jaglin et al. 2009; Kumar et al. 2010; Tischfield et al. 2010). Non-pathogenic *TUBB3* null alleles have been reported, supporting altered- or gain-of-function mechanisms for the missense variants. We previously reported that heterozygous missense variants in *TUBB3*, the gene that encodes the neuron-specific beta-tubulin isotype 3 (TUBB3, MIM: 602661), cause isolated or syndromic congenital fibrosis of the extraocular muscles type 3A (CFEOM3A, MIM: 600638), with remarkable genotype/phenotype correlations (Tischfield et al. 2010; Whitman et al. 2016). CFEOM is a rare, complex congenital cranial dysinnervation disorder (CCDD) caused by cranial nerve misguidance, particularly of the oculomotor nerve. The condition is characterized by non-progressive partial ophthalmoplegia, usually with ptosis. Vertical eye movements are severely limited while horizontal eye movements are variably limited. Of the TUBB3 amino acid substitutions that cause CFEOM, four (Arg62Gln, Arg262Cis, Ala302Thr, Asp417Asn) are often inherited as an autosomal dominant condition, while six others (Gly71Arg, Gly98Ser, Arg262His, Arg380Cis, Glu410Lys, Asp417His) are more likely to arise *de novo* and be associated with more severe neurological phenotypes (Tischfield et al. 2010; Whitman et al. 2016). A separate set of missense mutations in *TUBB3* cause malformations of cortical development (MCD) along with other brain abnormalities, but without CFEOM3 (Poirier et al. 2010).

Chew et al. (2013) described in detail the TUBB3 E410K syndrome that results from a *TUBB3* c.1228G>A missense variant (NM_006086.4; Chr16:89935679 (hg38); Glu410Lys). Individuals who harbor this variant have severe CFEOM, facial palsy, midface hypoplasia, Kallmann syndrome (hypogonadotropic hypogonadism and anosmia), intellectual and social disabilities, and a progressive peripheral sensorimotor polyneuropathy not symptomatic until the third decade of life, and many also have vocal cord paralysis and cyclic vomiting (Chew et al. 2013). On neuroimaging, these subjects display white matter abnormalities, particularly of the corpus callosum and corticospinal tracts (Chew et al. 2013; Grant et al. 2019).

We report here the detailed clinical manifestations of a similar but distinct syndrome in 14 individuals from 13 unrelated families associated with a *TUBB3 c.*785G>A variant (NM_006086.4; Chr16:89935236 (hg38); Arg262His) which we refer to as the TUBB3 R262H syndrome.

## MATERIALS AND METHODS

### Subjects

Research participants were enrolled either as part of an ongoing genetic study of congenital cranial dysinnervation disorders at Boston Children’s Hospital, Boston, MA (clinicaltrials.gov identifier NCT03059420) or as part of the Study on Moebius Syndrome and Congenital Facial Weakness Disorders at the National Institutes of Health (clinicaltrials.gov identifier NCT02055248). The Institutional Review Boards at Boston Children’s Hospital (Boston, MA) and National Human Genome Research Institute, National Institutes of Health (Bethesda, MD) approved the study. Informed consent was obtained from each participant or legal guardian. Individual-level data was de-identified. Sharing of medical record information between collaborating sites was covered by an inter-institute collaborative reliance agreement. Studies were performed in compliance with US 45.CFR.46 and the Declaration of Helsinki. Specific consent for the publication of identifiable photographs was also obtained where indicated.

### Genetic analysis

Eight study participants had their variant identified through clinical testing via targeted, exome, or genome sequencing. Three study participants provided a blood sample from which genomic DNA was extracted using Gentra Puregene Blood Kits (Qiagen) and the *TUBB3* gene was sequenced as previously described (Tischfield et al. 2010). The genetics of three study participants were previously reported (Tischfield et al. 2010).

### Phenotypic analysis of non-NIH participants

Detailed medical and family histories and clinical and neuroimaging data were obtained from study participants, their physicians, and their medical records. Clinical MRIs were obtained on a variety of scanners, including 3T Siemens Skyra and 1.5T GE Optima. Sequences included: Sagittal T1 Magnetization Prepared Rapid Gradient Echo (MPRAGE); Axial T2; Coronal T2; Sampling perfection with application-optimized contrasts using different flip angle evolution (SPACE); and High-resolution T1.

### Phenotypic analysis of NIH participants

Five of the study participants (Subjects VII, X, XI, XII, XIII) underwent deep phenotyping at the NIH Clinical Center including consultations with clinical genetics, neurology, ophthalmology, audiology, craniofacial, rehabilitation medicine, as well as neurocognitive, audiometric, three-dimensional facial imaging analysis, neuroimaging and electrophysiological studies as detailed below.

#### Neurocognitive testing:

IQ testing was conducted using the Wechsler Preschool and Primary Scale of Intelligence (WPPSI-IV), Wechsler Intelligence Scale for Children (WISC-V), Mullen Scales of Early Learning, or Wechsler Adult Intelligence Scale (WAIS-IV) tests, depending on the age and functional level of the subject (Achenbach and Ruffle 2000; Burns et al. 2013; Le Couteur et al. 2008; Mayes and Calhoun 2008; Wechsler 2012). The Child Behavior Checklist was administered to evaluate for emotional and behavioral concerns and standard instruments for autism screening were employed, including the Autism Diagnostic Interview-revised (ADI-R) and Autism Diagnostic Observation Schedule-2 (ADOS-2) as indicated.

#### Ophthalmology:

Neuro-ophthalmology evaluations included detailed characterization of the ocular dysmotility phenotype, recorded the eye movement patterns, assessed visual acuity, visual field exam, contrast sensitivity, color vision and dilated fundus examination.

#### Audiology:

Comprehensive audiometric evaluations included ear-specific air conduction (250-8000 Hz) and bone conduction (250-4000 Hz) pure-tone thresholds at octave frequencies. Normal hearing was classified as thresholds ≤25 dB HL. Tympanometry and acoustic reflex threshold data were interpreted using published normative data (Silman and Gelfand 1981). Distortion product otoacoustic emissions (DPOAE) were evaluated in quarter octave bands from 842 to 7996 Hz, using an f2/f1 ratio of 1.2 and L1 and L2 of 65- and 55-dB SPL, respectively. Neurodiagnostic auditory brainstem responses (ABR) were obtained to broadband clicks presented at 95 or 85 and 0 dB nHL (control run) at 8.3 clicks per second using an Fz to ipsilateral and contralateral earlobe electrode montage and a grounding electrode placed at Fpz. Absolute and interpeak latencies for waves I, III, and V of the ABR were evaluated using normative data (Schwartz et al. 1989) and the presence of a cochlear microphonic was determined by comparing responses to opposite polarity click stimuli.

#### Craniofacial assessment and 3D photo surface quantitative analysis:

Comprehensive craniofacial assessments with three-dimensional (3D) photos were obtained using the 3dMD two-pod system (Atlanta, GA) or Planmeca ProFace system (Planmeca, Roselle, IL) (Liberton et al. 2019). Each individual was captured with a neutral expression; the mouth was slightly open due to normal posture related to facial muscle weakness. The images were automatically stitched using respective proprietary software and exported in Wavefront OBJ (.obj) format with texture. No surface smoothing or hole filling was conducted, and quality checks were performed to ensure uniform stitching. Images were imported into MeshLab (Cignoni et al. 2008), where 24 landmarks were manually annotated by a trained observer. Facial landmarks and normative measurements were obtained from FaceBase 3D Facial Norms (www.facebase.org, NIDCR Project 1U01DE020078)(Brinkley et al. 2016). Landmark coordinates were exported in .csv format and 29 linear distances were measured. In one case, the landmark “Right Tragion” was missing, and the measurements associated with it were omitted from the analysis. As the open-mouth posture is associated with this condition, facial length measurements include the distance between upper and lower lips. Linear distances were compared to the appropriate sex- and age-matched norms to calculate a Z-score, which is the number of standard deviations from the norm. A Z-score of +/− 2 or greater was considered a clinically significant alteration of facial features; a Z-score between +/− 1-2 was considered a subclinical alteration. Normative data is only available for individuals of European/Caucasian ancestry and for individuals from 3-40 years of age.

#### Magnetic resonance imaging (MRI):

MRIs were obtained on 1.5T or 3T Philips Achieva scanner. Sequences included: Coronal T2 fluid-attenuated inversion recovery (FLAIR); Sagittal 3D T1 turbo field echo sensitivity encoding (TFE SENSE); Reformatted coronal 3D T1 TFE SENSE; Axial T2 3D volume isotropic turbo spin-echo acquisition (VISTA) constant level appearance (CLEAR). Axial 3D Faster brainstem CLEAR. Coronal short tau inversion recovery (STIR). Sagittal 3D fast imaging employing steady-state acquisition (FIESTA). Axial T2 constant level appearance (CLEAR).

#### Electrophysiological studies:

Electrodiagnostic studies were performed on Viking Select or EDX machines (Natus Medical Inc., Middleton, WI) as reported previously (Lehky et al. 2021). All five subjects enrolled at the NIH underwent median and peroneal motor nerve conduction studies (MNCs) and median and sural sensory nerve conduction studies (SNCs) using standard techniques. Three also underwent facial motor nerve conduction studies, recording from orbicularis oculi and orbicularis oris, and blink studies, and two underwent needle EMG of the limbs. Normative values were per published and lab normative tables (Preston and Shapiro 2013). These data were also included in Lehky et al. (2021), comparing them to individuals with other forms of facial weakness.

## RESULTS

### Subjects and Genetic Findings:

Each of fourteen affected individuals from thirteen unrelated pedigrees harbored a heterozygous *TUBB3* c.785G>A variant (p.Arg262His). The subjects currently range from infancy to 25 years of age and are of a variety of ethnic backgrounds (see Table 1). None of the 14 subjects had a family history of the TUBB3 R262H syndrome. All parents are clinically unaffected and, in the 11 cases (10 pedigrees) in which parental DNA was analyzed, the proband’s variant arose *de novo.* Subjects XI and XII are monozygotic twins with a *de novo* mutation; they and subject XIV were originally reported in (Tischfield et al. 2010) and updated clinical information since the time of that publication is included here.

### Pre- and Perinatal Findings:

Ten subjects had signs of fetal distress prior to birth, leading to premature delivery in three. Oligohydramnios was reported in one subject, and three were born small for gestational age or had intrauterine growth restriction. Birth weights are listed in Table 1.

All fourteen subjects presented as neonates with a complex neurological syndrome characterized by profound bilateral limitation of eye movements, large exotropia, and facial weakness. Thirteen were noted to have distal joint contractures at birth. Eight had vocal cord paralysis with perinatal distress and required tracheostomy placement shortly after birth. In a ninth subject, tracheostomy placement was recommended but the parents declined; at age 1 he continued to require continuous monitoring and frequent suctioning. Nine subjects required nasogastric or gastrostomy tube feeding during infancy. Two subjects had congenital unilateral sensorineural hearing loss.

Initial clinical diagnoses (prior to genetic diagnosis) included atypical Moebius syndrome in several subjects, Cary-Fineman-Ziter syndrome in one subject, and Marden Walker syndrome in two subjects.

### Growth:

Failure to thrive and short stature were common. Eleven subjects are below average for height and 13 subjects are below average for weight at their most recent measurements. Height and weight Z-scores range from −5.69 to +2.96 and from −7.76 to +0.39, respectively. (Table 1). BMI Z-scores ranged from −5.06 to +1.84 at the time of evaluation. Six subjects were noted to have microcephaly.

### Craniofacial:

**Fig. 1:**
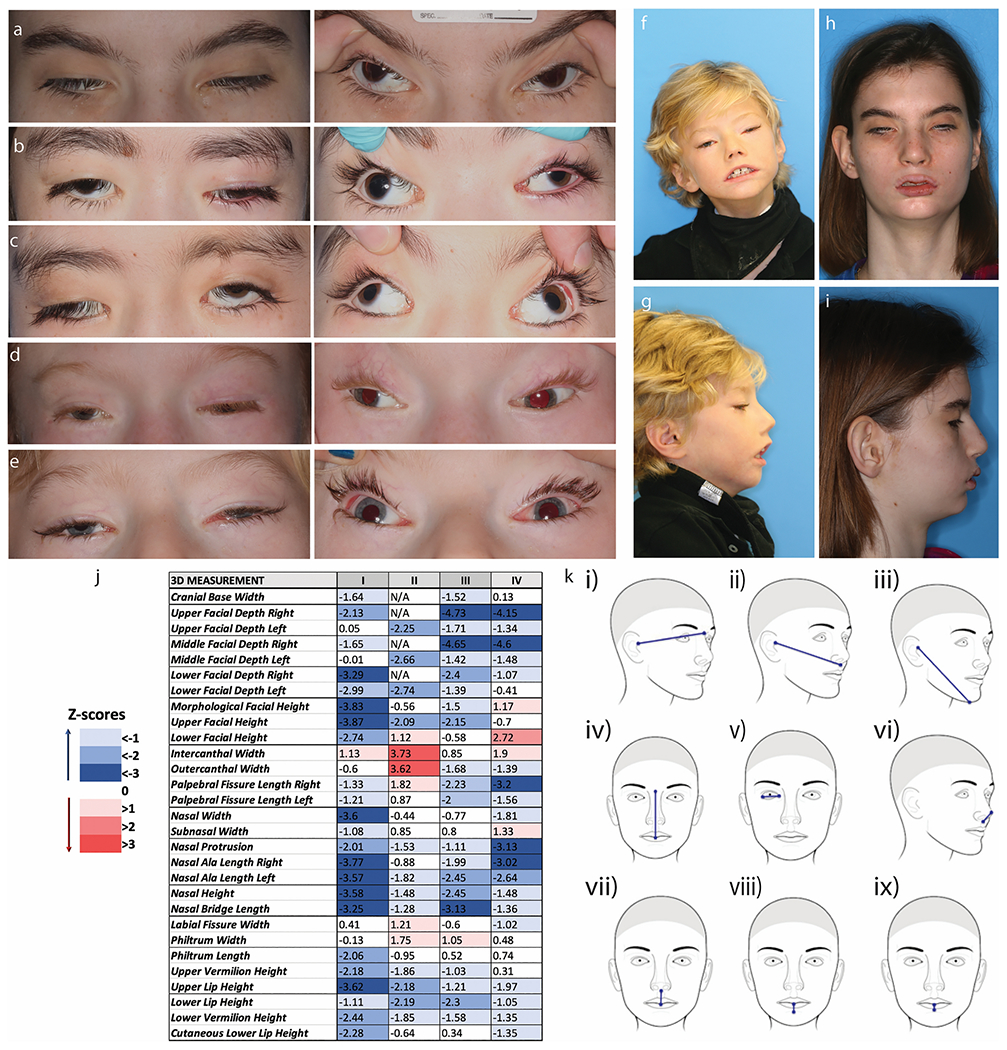
Ophthalmological and Craniofacial Evaluation. All subjects have severe ptosis, extremely limited eye movements, and severe exotropia. a-e Ocular photographs of the five subjects evaluated at NIH (XIII, X, XI, XII, VII). Panels on the left show the severe ptosis. Panels on the right, with the lids artificially raised, display the severe exotropia. Subjects have almost no voluntary eye movements. F-I. Frontal and profile photos of subjects VII (f&g) and XIII (h&i). j. Color scale table depicting the deviation of the soft-tissue measurements in four TUBB3 R262H subjects, expressed in the form of Z-scores, which were computed from the normal values provided by FaceBase. k. Schematic representation of the most negatively affected linear 3D measurements in this cohort. In general, most values are lower than the average normal values, particularly the facial depth measures (i, ii, iii), the upper facial height (iv), the palpebral fissure lengths (v), the protrusion of the nose (vi), and the vertical dimensions of the lips (vii, viii, ix). The only measure with increased or normal values in all four subjects is the intercanthal width.

All subjects have facial dysmorphology and facial weakness. Four subjects were evaluated with 3D photography at NIH. Quantitative analysis of the 3D photos shows morphological similarities, despite the phenotypic variability between the four subjects (Figure 1). Most of the assessed craniofacial measures had values below the norm, suggesting a smaller overall face size despite the open-mouth posture (potentially lengthening the face), and under-projection of the facial structures of these subjects when compared to age- and sex-matched controls. Measurements of facial depth, nose protrusion, upper facial height, vertical lip dimensions, and palpebral fissure length were significantly reduced in the cases quantified. Only intercanthal width measurements were normal or increased in the four subjects quantified.

### Joint contractures:

**Fig. 2:**
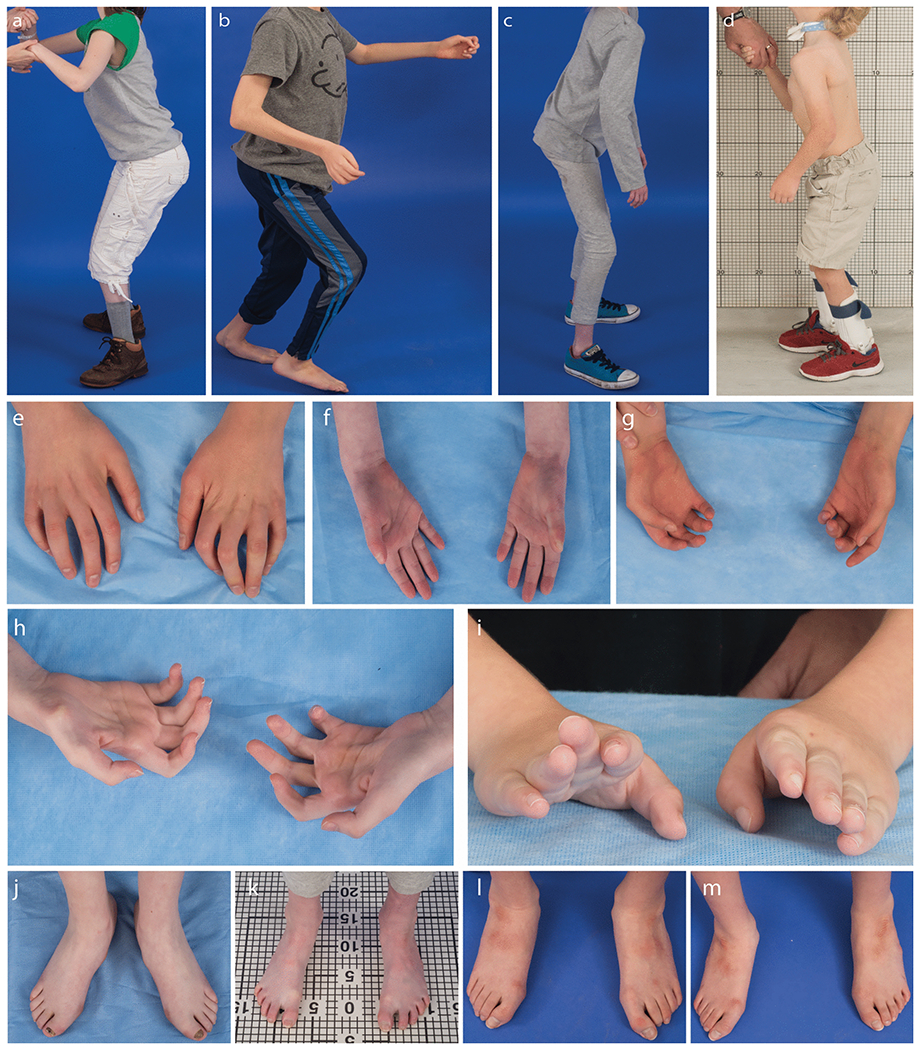
The combination of joint contractures and peripheral neuropathy limit mobility. Standing photographs of subjects XIII (a), XI (b), X (c), and VII (d) show the wide based, hinged stance subjects require to stand. Representative images of hand (e-i) and feet (h-m) contractures from subjects VII (i), X (f,k), XI (g,m), XII (e,l), and XIII (h,j).

Thirteen subjects have congenital and one has acquired contractures of distal joints of varying severity. The distal contractures often worsened and more proximal contractures developed over time (Figure 2). Subjects I, II, and V have congenital camptodactyly. Subject I also has unilateral talipes equinovarus (clubfoot), and Subject II had bilateral mild foot adduction and long 1st and short 2nd-5th metatarsals. Subject III has congenital hand and foot contractures requiring physical therapy, hand braces, lower extremity casting, and foot surgeries. Subject IV has bilateral congenital ulnar wrist deviation. Subject VI has congenital ankle and toe contractures requiring lower extremity casting. Subject VII has congenital contractures of the elbows and knees, clawed toes, clinodactyly, and fisted thumbs/hands that required splinting and gloves as an infant, developed coxa valgus and genu varus, and underwent proximal tibial epiphysiodesis for leg length discrepancy. Subject VIII has congenital contractures of the upper and lower extremities and was diagnosed with arthrogryposis multiplex congenita. Subject IX has congenital coxa vara deformity of the hip. Subject X had short thumbs without contractures at birth and developed upper limb hypermobility, progressive bilateral hamstring and Achilles contractures, and a leg length discrepancy. Subject XI had congenital contractures of the upper and lower extremities, hypoplasia of the thenar eminence, syndactyly, clinodactyly, and pes planovalgus. He also developed progressive hamstring and Achilles contractures and a leg length discrepancy. Subject XII has congenital finger contractures with clenched hands, and developed upper limb hypermobility, “windswept” hips, and progressive hamstring and Achilles contractures. Subject XIII has congenital progressive bilateral Achilles contractures and developed progressive finger and hamstring contractures and genu valgum. Subject XIV has congenital progressive hand and foot contractures.

Substantial physical therapy starting in infancy has been associated with the best motor function outcomes. Subjects have had a variety of orthopedic procedures and casting to address joint contractures, with varying levels of success. Some procedures have brought about functional improvements but, for other subjects, prolonged postoperative periods of immobility led to functional losses that were not regained.

### Neurodevelopment and neuroimaging:

Ten subjects are left-handed; of the remaining four, three are too young to display handedness and one (XIV) is noted to have poor use of both hands. All fourteen subjects have varying degrees of global developmental delay, better speech comprehension and communication skills compared to other domains, and pleasant and engaging personalities. Among those tested, all had low scores on the adaptive behavior composite (Table 2). Among the highest functioning subjects, subject X has a measured IQ in the average normal range with specific learning disorders and ADHD, and subject XIII had a measured IQ in the borderline range. Both have fluent language skills, with higher performance in areas of verbal comprehension and visual-spatial and fluid reasoning and lower performance in areas of working memory and processing speed when visual stimuli were introduced. Subject VI, whose IQ is not known, has strong language skills and did not qualify for early elementary special education services, but is notably weaker in mathematics. Among the lower functioning subjects, both monozygotic twins (XI and XII) carry a diagnosis of autism spectrum disorder, and one is completely dependent for activities of daily living. Subject XIV is also severely impaired and lives in a group home for intellectually disabled adults. One subject had partial, primarily subclinical, seizures requiring antiepileptic therapy in infancy, one subject has myoclonic movements during sleep for which she takes antiepileptic medication, and one has an epileptic focus in the temporal lobe requiring ongoing therapy; none of the other subjects has seizures.

**Fig. 3:**
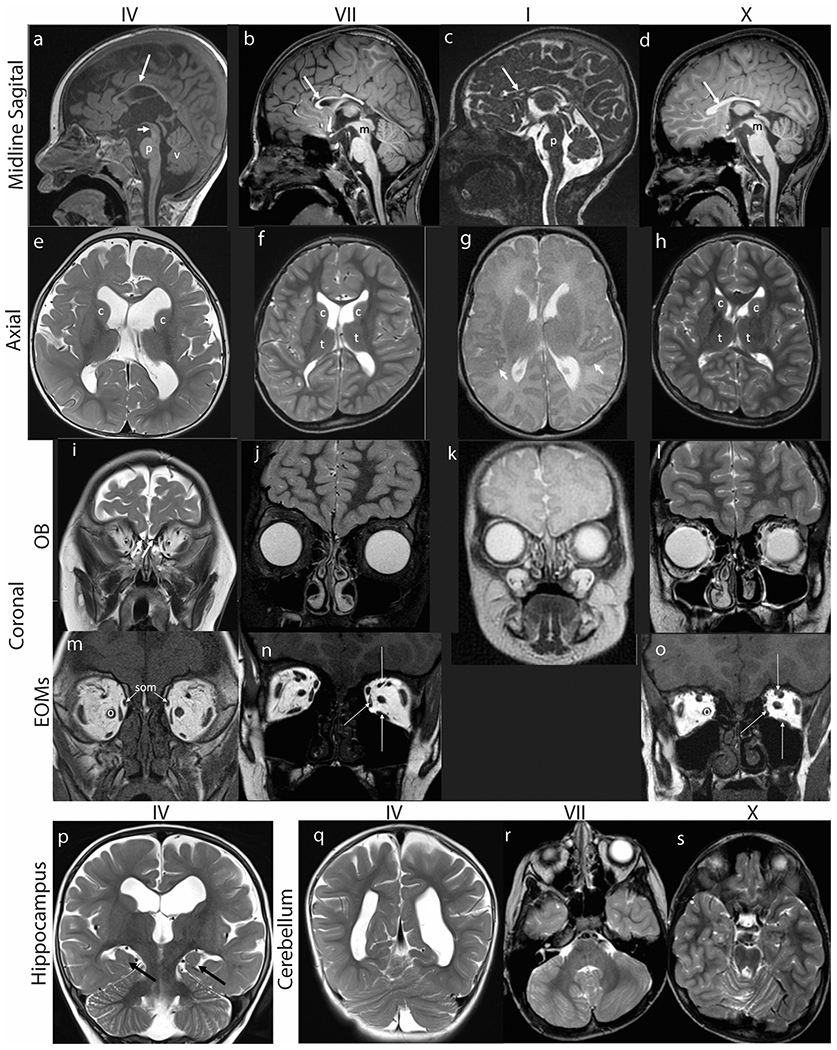
Brain MR imaging. Hypoplasia of corpus callosum, dysmorphic basal ganglia, absence of olfactory bulbs, small extraocular muscles, incomplete hippocampal inversion, and cerebellar abnormalities in subjects IV (a,e,i,m,p,q), VII (b,f,j,n,r), I (c,g,k) and X (d,h,l,o,s). (**a-d**) Midline sagittal T1 weighted (a, b, d) or FIESTA (c) images demonstrate marked thinning of the corpus callosum (long arrows). (**a**) The pons (p) appears mildly shortened in height and narrowed in AP diameter. The tegmentum of the midbrain (short arrow) appears slightly thinned and elongated. There is slight uplifting of the vermis (v). (**b**) The pons is minimally narrowed in AP diameter with slightly accentuated dorsal indentation and the distal midbrain (m) tegmentum is thinned. (**c**) The pons (p) is hypoplastic, appearing shortened in height and anteroposterior diameter with accentuated dorsal indentation. The vermis is slightly uplifted but normal in size. (**d**) There is mild thinning of the distal midbrain (m) and the pons is minimally shortened in height with accentuated dorsal indentation. (**e-h**) Axial T2 weighted MR images of the brain demonstrate asymmetry of the lateral ventricles and dysmorphic basal ganglia. (**e**) The caudate nuclei (c) and basal ganglia are asymmetric in shape and position, with the left caudate head more dorsally positioned than the right. The white matter appears mildly decreased in thickness. The lateral ventricles are prominent. (**f**) The caudate heads (c) are asymmetric with the left appearing shorter and wider. Mildly asymmetric shape and positioning of the thalami (t). The Sylvian fissures are asymmetric with the left shorter in length than right. The parietal and right frontal white matter appears mildly decreased in thickness with a small focus of high signal intensity adjacent to the right lateral ventricular atrium. (**g**) The perisylvian cortex has a serrated appearance raising concern for polymicrogyria (short arrows). (**h**) The lateral ventricles, caudate heads (c) and thalami (t) are asymmetric in shape. Mildly decreased thickness of right frontal and left > right parietal periventricular white matter. (**i-l**) Coronal MR images at the level of the cribriform plates (white arrows in I) reveal absent olfactory bulbs and sulci with only small vessels visible. (**m-o**) Coronal T1 weighted MR images show small medial, superior, and inferior rectus muscles (long white arrows in N and O). The superior oblique muscles (som, arrows, M) are also small and inferomedially positioned. The optic nerves (o) appear medialized. (**p-q**) Coronal T2 weighted MR images of the brain show (P) incomplete hippocampal inversion bilaterally (black arrows). The 3^rd^ and lateral ventricles are moderately dilated with the left lateral ventricle appearing larger than the right. The body of the left caudate nucleus appears smaller than the right (c), and more inferolaterally positioned. (**q**) The cerebellum appears mildly rotated. (**r**) Axial T2 weighted MR image at the level of the posterior fossa shows asymmetric architecture of the cerebellar hemispheres with generalized mild dysmorphism on the right. There is minimal disorganization of the superior cerebellar vermian folia. (**s**) Axial T2 weighted images show minimal dysmorphism of the superior cerebellar vermis.

**Fig. 4:**
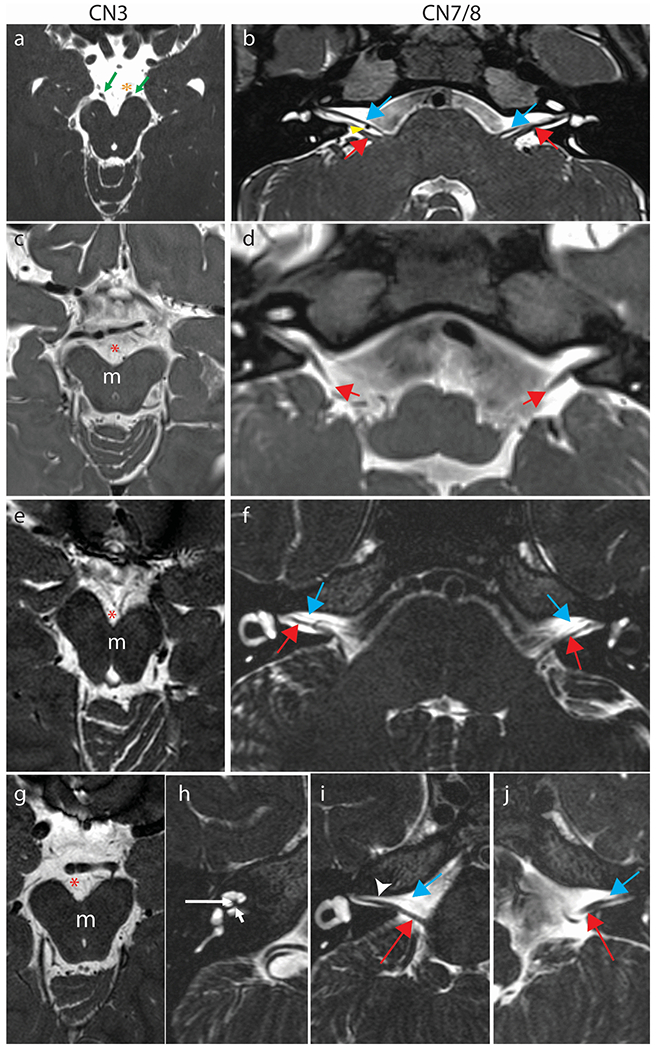
Cranial nerve MR imaging. Oculomotor nerve (a,c,e,g) and facial nerve (b,d,f,i,j) and cochlear nerve (h) imaging in comparison subjects (a,b), Subjects IV (c,d), X (e,f), and VII (g-j). (**a**) Normal CN III. 3 Tesla Axial constructive interference in steady-state (CISS) image through the midbrain at the level of the interpeduncular cistern showing normal CNs III emerging from the ventral aspect of the cerebral peduncles (green arrows). The oculomotor nerves course inferolaterally to the cavernous sinuses in close proximity to the posterior cerebral artery (PCA) on each side (orange asterisk left PCA). (**b**) Normal CN VII and VIII. 3 Tesla Axial sampling perfection with application optimized contrasts using different flip angle evolution (SPACE) image demonstrating normal anatomy of CN VII (blue arrows) coursing parallel and ventral to CN VIII (red arrows) on each side. The anterior inferior cerebellar artery flow void is seen between the cranial nerves on the right (yellow arrowhead). (**c,e,g**) Axial sampling perfection with application optimized contrasts using different flip angle evolution (SPACE) images (c) or Axial T2 weighted (e,g) images at the level of the interpeduncular cistern (red asterisk) show apparent absence of the cisternal segments of cranial nerves III which usually course anterolaterally from the ventral aspect of the midbrain (m) on each side. (**d,f,i,j**) At the level of the superior internal auditory meati, the cisternal segments of cranial nerve VIII are well seen bilaterally (red arrows, d [SPACE], f, i, j [axial T2 weighted]). Cranial nerves VII, which ordinarily course anterior and parallel to VIII are either not seen on either side (d) or appear markedly hypoplastic (blue arrows in f, i, j) and are faintly visualized coursing ventral and parallel to the cranial nerves VIII (red arrows). The right internal auditory meatus is asymmetrically narrowed (i, arrowhead). (**h**) In subject VII, who has right-sided hearing loss, the cochlear modiolus is thickened and misshapen (longer white arrow) with severe stenosis/atresia of the cochlear aperture (short white arrow). The cochlear division of the right CN VIII, which usually courses through the cochlear aperture, is not visible.

Structural brain neuroimaging revealed marked thinning of the corpus callosum in all subjects. Eight subjects had high-quality imaging (Figures 3–4, Table 3). Additional common findings included asymmetrical dysgenesis of the basal ganglia and adjacent lateral ventricles, absence of the olfactory bulbs and sulci, asymmetrically dysmorphic superior cerebellar hemispheres, and small extraocular muscles (Figure 3). Three subjects had Sylvian fissure asymmetry; in subject I there was associated polymicrogyria (Figure 3G, arrows). Three subjects had abnormal rotation of the hippocampi (Figure 3). Four subjects had decreased white matter volume and the pons appeared small and slightly dysmorphic in four subjects. Relevant cranial nerve imaging is detailed below.

### Cranial Nerve Function and Imaging:

The subjects have dysfunction of multiple cranial nerves.

#### Olfactory function:

Anosmia/hyposmia is reported in six subjects, although only two (XIII and X) completed formal testing. None of the subjects who subjectively report normal sense of smell completed formal olfactory testing.

#### Optic nerve function:

Three subjects have minimally reactive and asymmetric pupils and two others have small optic nerves (Table 3). All have moderately decreased vision due, at least in part, to amblyopia and corneal exposure.

#### Oculomotor, trochlear, and abducens nerve function:

All subjects have CFEOM with severe bilateral, often asymmetric findings of ptosis, hypotropia, large-angle exotropia, and minimal to no residual vertical or horizontal eye movements (Figure 1, Table 1). Six subjects have a history of strabismus surgery; results were generally disappointing, with recurrence of large exotropia in all subjects. Seven subjects have a history of ptosis surgery (frontalis sling). Several subjects were noted to use their fingers to lift their eyelids when looking at something. In all subjects with cranial nerve imaging, the oculomotor nerve (cranial nerve 3) was not visualized (Figure 4).

#### Facial nerve function:

All fourteen subjects have bilateral facial weakness. Three subjects had facial motor nerve conduction (MNC) and blink studies performed. All three had low amplitude MNC responses in at least two muscles (varied by side and which muscle) and no recordable blink response (Table 2). In all subjects with cranial nerve imaging, the facial nerve (cranial nerve 7) was severely hypoplastic or could not be visualized (Figure 4).

#### Auditory nerve function:

Two (VII, XIII) of the four subjects who underwent audiological assessments had unilateral congenital sensorineural hearing loss ranging from moderate to profound in degree. In both ears with hearing loss, otoacoustic emissions and ABRs were absent with large cochlear microphonics, suggesting both cochlear and eighth nerve pathology. This is supported by MRI studies indicating severe hypoplasia of the auditory nerve (VII, XIII) and cochlear abnormalities (VII) (Table 3, Figure 4) for the ears with hearing loss. Additionally, ABRs for the normal hearing ears of these two individuals exhibited signs of neural dys-synchrony. The other two subjects tested (XI and XII) had normal hearing sensitivity in both ears; however, ABRs also suggested neural dys-synchrony (e.g., present waveforms with poor morphology) bilaterally. Overall, all eight ears provided evidence supporting some degree of neural dys-synchrony, despite the presence of normal peripheral hearing sensitivity in six ears.

#### Glossopharyngeal nerve function:

Ten subjects required tube feeding in infancy and nine have ongoing swallowing difficulties.

#### Vagus nerve function:

Nine subjects had vocal cord paralysis. Eight had tracheostomy placement within the first months of life, which was removed in the three oldest subjects between 7-10 years of age. The five subjects who still have a tracheostomy range from infancy to 13 years. Tracheostomy removal has not been attempted in subject VII (age 13).

### Motor Development and Function:

Gross motor milestones were mildly to markedly delayed in all subjects and all of ambulatory age have required use of Ankle Foot Orthoses (AFOs). Of the nine subjects over age 5, four learned to walk unassisted and five learned to walk with assistive devices; motor function has generally worsened by adolescence. Subjects VI and X are the least motorically impaired and learned to walk independently without AFOs at 18 months of age. Both are currently able to walk and play sports, but VI cannot walk long distances and X has frequent falls and is now using AFOs. Subject VII learned to walk with AFOs and a walker at age 3; he continues to require support when walking. Subject XI walked with support at age 3 and unassisted at age 6, but motor function worsened with age; he now requires AFOs and a walker for community ambulation. Subject XII is the most motorically impaired; he learned to stand independently at age 3, but has had significant decline in functional mobility and now needs a walker or wheelchair at all times. Subject XIII walked with AFOs at age 3; she is now able to ambulate short distances unsupported, but has frequent falls and prefers a wheelchair in many settings. Subject XIV was able to walk with support until 8 years of age when he underwent foot surgery. Following surgery, he never regained the ability to walk.

### Motor Tone and Strength:

Although all 14 subjects were reported at some point to have hypotonia, the five subjects evaluated at NIH were found to have normal axial and appendicular tone with upper limb hypermobility. Subject VII displayed full muscle strength throughout. Subject X had moderate hip girdle weakness. Subjects XI and XII could not cooperate with strength testing; both seemed to have proximal weakness greater in the lower compared to upper limbs, and XII had decreased muscle bulk. Subject XIII had significant hip girdle and lower extremity flexor and extensor weakness, with extensor hallucis longus strength of only 1/5 bilaterally.

### Reflexes:

In the four subjects tested, deep tendon reflexes were decreased (XI), increased (XIII) or normal (VII, XII), and the Babinski reflex was positive/extensor with ankle clonus (X, XII, XIII) or negative/flexor (VII). These findings are consistent with the combination of spasticity and peripheral neuropathy.

### Sensory:

Subject VII reports very high pain tolerance in his feet and decreased sensation in the lower extremities, with normal sensation in the upper extremities. Subject XIII has decreased perception of 5.07 monofilament in a stocking distribution in the feet to just above the ankles.

### Electrodiagnostic testing:

The eight subjects who have undergone nerve conduction studies all had abnormal initial studies, regardless of age; the youngest tested was Subject VIII who had an abnormal study at one year of age. Five of the subjects underwent nerve conduction studies and two underwent needle EMG studies at NIH (at ages 7, 12, 14, 14 and 19) (Table 2). Peroneal motor nerve conduction studies (MNCS) revealed no recordable response when recording from the extensor digitorum brevis in 4 subjects, while one had low amplitude responses. Median MNCs revealed low amplitude responses in 3 and normal amplitude responses in 2 subjects. Sensory nerve conduction studies (SNCS) of the sural and median nerves revealed no recordable response in 3 subjects and low amplitude responses in 2 subjects. All conduction velocities were slow and in the demyelinating range. In 19-year old subject XIII, needle EMG of the right vastus lateralis and tibialis anterior showed mild active denervation, decreased recruitment, and large motor units consistent with a chronic neurogenic process in the leg muscles. By contrast, the right biceps brachii, a proximal arm muscle, was within normal limits. In 7-year old subject VII, a needle EMG of the tibialis anterior was within normal limits, likely reflecting less progression of neuropathy at the younger age.

### Endocrine:

Kallmann syndrome is characterized by impaired sense of smell and absent or delayed puberty secondary to hypogonadotropic hypogonadism. The diagnosis is supported by micropenis and/or cryptorchidism in infant males and by absence or hypoplasia of the olfactory bulbs and olfactory sulcus on MR imaging. All subjects display one or more manifestations of Kallmann syndrome but many are too young for a definitive diagnosis. Of the nine males, six were noted at birth to have micropenis and/or cryptorchidism (signs of hypogonadotropic hypogonadism); none received hormonal treatment in infancy. The eight subjects with informative MR imaging had absence or hypoplasia of the olfactory bulbs and olfactory sulcus. Both of the monozygotic twins have an absence of olfactory bulbs on neuroimaging; one had delayed puberty with abnormal laboratory testing (LH <0.1 U/L, FSH 0.4 U/L, total testosterone <20.0 ng/dl), while the other had normal onset of puberty and normal laboratory testing (LH 3.0 U/L, FSH 4.9 U/L, total testosterone 191ng/dl). The three females who have reached young adulthood have anosmia and hypogonadotropic hypogonadism based on lack of pubertal signs, primary amenorrhea, and laboratory studies. Male subjects have required testosterone supplementation and female subjects have required estrogen to induce puberty followed by estrogen and progesterone supplementation. When available, bone density studies in both males and females showed decreased bone density and delayed bone age. Bone health is supported with dietary calcium, vitamin D monitoring and supplementation as needed, and physical activity with weight-bearing exercise.

### Cardiac:

The oldest subject (Subject XIV) underwent pacemaker placement at 21 years of age for treatment of sinus node dysfunction and sinus arrest pauses up to 7.5 seconds, causing syncopal episodes. ECG revealed a right bundle branch block and echocardiogram revealed no structural cardiac abnormality. Ten other subjects were noted to have asymptomatic elevated resting heart rates ranging from 110-150 beats per minute noted incidentally both on initial physical examination and on repeated vital sign documentation. The tachycardia has resolved in one subject. Among the nine subjects currently with tachycardia, those tested have had normal ECGs and echocardiograms and none report cardiac symptoms, exercise intolerance, or need for any cardiac intervention.

### Respiratory:

Eight subjects required tracheostomy due to vocal cord dysfunction, but all have normal respiratory function and are not ventilator dependent. The older subjects were successfully decannulated by 10 years of age, with the exception of Subject VII, now 13 years old, for whom decannulation has not been attempted.

### GI:

One subject (VII) required Nissen fundoplication in infancy. Three subjects have cyclic vomiting syndrome (Table 1). Subject VIII has required hospitalization for vomiting episodes, which began around age 7, caused by respiratory infections or stress and has had a good clinical response to ondansetron. Subject IX has had multiple hospitalizations for vomiting, starting at age 11, and has had a good clinical response to pizotifen. Subject X has had several episodes of cyclic vomiting, starting at age 15. She has required hospitalization for vomiting and aspiration pneumonia, and eventually it was determined her vomiting could be alleviated with haloperidol.

### Renal:

One subject (IX) developed marked proteinuria and rapidly progressive, ultimately fatal, chronic kidney disease at age 15, with normally positioned but small and echogenic kidneys. Kidney biopsy was not obtained. While it remains unknown whether this was related to TUBB3 Arg262His, no other subjects have a history of renal disease, and at least three (XI, XII, XIII) have had normal renal ultrasounds.

## DISCUSSION

The TUBB3 protein is a component of microtubules expressed in all neurons of the developing and mature central and peripheral nervous system, including sympathetic, parasympathetic, and enteric neurons (Jiang and Oblinger 1992; Katsetos et al. 2003). Heterozygous missense variants in *TUBB3* cause a wide variety of phenotypes, ranging from lethal cortical malformations to isolated eye movement disorders, with very clear genotype-phenotype correlations. Here, we show that the TUBB3 Arg262His substitution presents as a distinct, clinically identifiable syndrome and is the most debilitating among the CFEOM-TUBB3 syndromes defined to date. The TUBB3 R262H syndrome includes CFEOM3, congenital facial weakness, intellectual disabilities, Kallmann syndrome, joint contractures, motor disabilities, and progressive peripheral neuropathy with onset in the first decade of life. Most affected individuals have congenital contractures and some have auditory dysfunction, vocal cord paralysis, swallowing difficulty, and cyclic vomiting. No inherited cases of this substitution have been identified, indicating that affected individuals may have reduced fecundity from neurological deficits and/or hypogonadotropic hypogonadism. Subjects with TUBB3 R262H syndrome received a variety of other diagnoses prior to their genetic diagnosis, including Moebius syndrome, Cary-Fineman-Ziter syndrome, and Marden Walker syndrome, all of which have some overlapping features with TUBB3 R262H. Table 4 compares the distinguishing features of each disorder.

Human *TUBB3* missense mutations have been reported that alter Arg262 to a histidine (Arg262His) or to a cysteine (Arg262Cys) (Tischfield et al. 2010), and both substitutions are predicted to disrupt a predicted hydrogen bond between Arg262 and Asp417. The phenotypes resulting from each of these mutations are, however, quite distinct. TUBB3 R262C syndrome subjects have an isolated CFEOM3 eye phenotype, with variable limits of vertical and horizontal eye movements, with or without ptosis. They have no other cranial nerve abnormalities or neurological signs or symptoms, and there are large, autosomal dominant pedigrees that segregate this variant (Tischfield et al. 2010).

The TUBB3 R262H syndrome is similar to but more debilitating than the second-most-severe TUBB3-CFEOM substitution, Glu410Lys, that results in the TUBB3 E410K syndrome. Both syndromes include severe CFEOM, developmental delays, facial weakness, a progressive peripheral neuropathy, and Kallmann syndrome, with vocal cord paralysis and cyclic vomiting in a subset (Chew et al. 2013). The cases evaluated to date, however, permit the two syndromes to be distinguished clinically. Those with the R262H syndrome have congenital, progressive, and acquired joint contractures, and onset of the progressive peripheral neuropathy typically prior to age 12. As a result, subjects have significant limitations in mobility and ambulation. By contrast, those with the E410K syndrome do not have joint contractures or auditory dysfunction, they walk unassisted, and they have a later onset of their progressive peripheral neuropathy in the late first or second decade of life (Table 4). The R262H and E410K syndromes can also be distinguished from one another by brain MR imaging. Both syndromes result in hypoplasia of the corpus callosum and/or anterior commissure, decreased white matter volume, absence of the olfactory bulbs and sulci, absence or hypoplasia of oculomotor and facial nerves, and small extraocular muscles (Chew et al. 2013; Grant et al. 2019). The R262H but not E410K syndrome, however, also results in dysmorphic basal ganglia and lateral ventricles, and often mildly dysmorphic superior cerebellum, abnormal rotation of the hippocampus, and small or dysmorphic pons. One R262H syndrome subject, the youngest, also has polymicrogyria of the perisylvian cortex, and two other subjects have asymmetry of the sylvian fissures. Malformations of cortical development (MCD) without CFEOM3 are reported to result from a distinct set of amino acid substitutions in TUBB3 (Porier et al 2010), and only two amino acid substitutions (Gly71Arg and Gly98Ser) have previously been reported to cause both CFEOM3 and MCD (Whitman et al. 2016).

*In vitro* and *in vivo* studies of a series of *TUBB3*-CFEOM amino acid substitutions, including Arg262His, support a dominant altered-function mechanism in which mutant tubulin is incorporated into the neuronal microtubule and disrupts specific aspects of motor protein–based axonal transport (Minoura et al. 2016; Niwa et al. 2013; Tischfield et al. 2010). TUBB3 harboring amino acid substitutions Arg262His, Glu410Lys, or Asp417His, which cause the more severe CFEOM-TUBB3 phenotypes, incorporate into microtubules, alter microtubule dynamics, and disrupt kinesin binding (Minoura et al. 2016; Niwa et al. 2013; Tischfield et al. 2010). By contrast, TUBB3 Arg262Cys, which causes the most mild phenotype, reduces the yield of native heterodimers by in vitro transcription and translation and incorporates poorly into HeLa cells compared to Arg262His and Glu410Lys (Tischfield et al. 2010). Consistent with these findings, complete loss of *Tubb3* in mice does not cause a developmental phenotype (Latremoliere et al. 2018) while introduction of a homozygous *Tubb3* Arg262Cis substitution results in defects in the growth and guidance of commissural axons and cranial nerves (Tischfield et al. 2010). Such studies have led to the hypothesis that the greater the level of mutant heterodimer incorporation into microtubules, the more severe the human phenotype (Tischfield et al. 2010). It remains unknown if subtle differences in incorporation between Arg262His, Glu410Lys, and Asp417His or other effects of these specific substitutions account for their phenotypic differences.

The TUBB3 Arg262His and Asp417His substitutions are the only TUBB3 substitutions reported thus far to cause congenital hand and foot joint contractures. *TUBB3* is expressed in neurons and their processes and not in muscle, connective tissue, or bone, and so these contractures most likely arise from *in utero* spinal motor neuron dysfunction unique to this most severe CFEOM-TUBB3 syndrome. Notably, distal arthrogryposis type 5 can be associated with blepharophimosis, ptosis and ophthalmoplegia (Sahni et al. 2004), and one genetic cause is biallelic variants in the endothelin-converting enzyme-like 1 (*ECEL1*) gene (Dieterich et al. 2013; McMillin et al. 2013; Shaheen et al. 2014). Subjects with *ECEL1* variants display congenital distal arthrogryposis together with variably penetrant combinations of ptosis, strabismus, and ophthalmoplegia, similar to subjects with the TUBB3 R262H syndrome. In some subjects, the ocular phenotypes are more prominent and the joint contractures do not meet the full diagnostic criteria for distal arthrogryposis (Khan et al. 2014; Shaaban et al. 2014; Shaheen et al. 2014). ECEL1 is a member of the neprilysin family of zinc metalloendopeptidases implicated in the final branching of axon nerve terminals and formation of neuromuscular junctions (Kiryu-Seo et al. 2019; Nagata et al. 2016; Nagata et al. 2017). The similarity of the phenotypes between subjects with *ECEL1* mutations and the TUBB3 R262H syndrome may reflect the participation of *ECEL1* and *TUBB3* in a common pathway of axon guidance to distal muscles.

Subjects with the R262H syndrome develop a sensorimotor polyneuropathy with an earlier age of onset but similar mixed axonal and demyelinating electrodiagnostic features as previously reported in the TUBB3 E410K and D417N syndromes (Chew et al. 2013; Hong et al. 2015). Needle electromyography was neurogenic when abnormal, and a previously reported sural nerve biopsy from an individual with a peripheral neuropathy secondary to the TUBB3 D417N syndrome was neurogenic (Hong et al. 2015). Finally, *TUBB3* is expressed in sensory and motor neurons but not in Schwann cells. Together, these data support an early-onset primarily axonal peripheral neuropathy with secondary demyelination.

Subjects with the TUBB3 R262H syndrome develop significant disabilities of posture and gait. Most likely, the congenital ankle and foot contractures, spasticity, and progressive peripheral neuropathy cause proximal and distal muscle weakness and poor joint alignment, leading to secondary proximal muscle contractures. Confounders such as growth with weight gain and inactive lifestyle can contribute to the need for assistive devices or wheelchair use. Subjects who were followed closely for development of contractures and treated early to improve joint alignment have had the best motor outcomes.

At least ten of the fourteen subjects are left-handed, well above the incidence of left-handedness in the general population. The neural mechanisms of establishing handedness are unknown, but it is interesting that a GWAS of handedness has identified multiple significant loci associated with genes involved in brain development and patterning, including microtubule-related *MAP2* and *MAPT* and the tubulin genes *TUBB, TUBB3, TUBB4A,* and *TUBA1B* (Cuellar-Partida et al. 2021; Wiberg et al. 2019). The specific mechanisms by which polymorphisms in any of these genes lead to left-handedness (and how TUBB3 Arg262His leads to left-handedness) are unknown.

Kallmann syndrome (hypogonadotropic hypogonadism with anosmia or hyposmia) results when disruption of olfactory migration also disrupts the migration of hypothalamic GnRH-producing neurons from their birthplace in the nose into the central nervous system along the olfactory/vomeronasal nerve. Interestingly, there is variable expressivity of the Kallmann phenotype with the TUBB3 Arg262His substitution; of the monozygotic twins in our study, one presented with normal puberty and the other with delayed puberty, despite both having absence of olfactory bulbs on neuroimaging.

One TUBB3 R262H subject required pacemaker implantation in young adulthood. Although not originally reported in TUBB3 E410K syndrome, we have subsequently learned of one E410K subject who had a history of infantile SVT and onset of syncopal events at age 17 secondary to sinus node dysfunction with pauses up to 25 seconds; the events resolved following pacemaker implantation (unpublished). An E410K subject has also been reported with cough syncope (Nakamura et al. 2018). While it has not been established whether these findings are caused by the *TUBB3* variants, together they raise the possibility that cardiac conduction problems are related to the TUBB3 peripheral neuropathy or autonomic dysfunction. Several other subjects have been incidentally noted to have asymptomatic sinus tachycardia with normal echocardiograms when available. While the tachycardia may simply reflect anxiety with medical procedures, it could also be an indication of future cardiac conduction problems. Therefore, additional cardiac and autonomic testing should be considered, particularly if symptoms such as syncope or near-syncope develop.

One subject developed renal disease, which proved fatal. Although renal anomalies have been reported in subjects with Kallmann syndrome (Bonomi et al. 2018; Zenteno et al. 1999), TUBB3 is not expressed in the kidneys, and there have not been reports of renal disease associated with tubulinopathies. It is therefore unclear whether her renal disease is related to her tubulin mutation.

## CONCLUSION

Despite the ubiquitous expression of *TUBB3* in neurons, different missense variants in *TUBB3* cause very specific neurologic deficits. The extent of the phenotypes and the neuronal populations involved are exquisitely linked to the specific amino acid substitution. We describe here the most severe form of syndromic CFEOM recognized to date, the TUBB3 R262H syndrome. These subjects require multidisciplinary care, including ophthalmology, orthopedics/physiatry, physical therapy, neurology, and endocrinology. Care must be taken to prevent and treat corneal exposure and amblyopia to maximize vision. Extensive physical therapy and treatment of joint contractures maximizes functional mobility. Endocrine intervention can induce puberty and bone density needs to be monitored and supported. These newly described phenotypes lead to better characterization of the spectrum and severity of disease associated with mutations in TUBB3, enhancing the accuracy of genetic testing and allowing for more tailored (personalized) medical care and genetic counseling.

## Figures and Tables

**Table 1:** Phenotypic Features m: months; CE: Caucasian of European ancestry; IUGR: intrauterine growth restriction; Wt: weight; Facial dysmor: facial dysmorphism; ID: intellectual disability; Clin PN: clinical peripheral neuropathy; EMG/NC abnl: electromyography or nerve conduction abnormal; JC: joint contractures; Ex: extremity; Olf: olfactory bulb; Cycl: cyclic; arryth: arrythmia; OD: right eye; OS: left eye; BCVA: best corrected visual acuity; A: anisocoria; XT: exotropia; ONH: optic nerve hypoplasia; NFA: normal for age; R: reactive; G: glaucoma; Ec: ectropion; En: entropion; Ab: aberrant ocular movements; ON: optic nerve, b/l: bilateral; hypo:hypoplasia; TY: too young to test; ND: not done; N/A: not applicable; U: unknown/not available.

Subject	I	II	III	IV	V	VI	VII	VIII	IX	X	XI	XII	XIII	XIV
Age Gender	3m M	18m M	18m F	2 M	5 M	8 F	12 M	14 M	16 F	17 F	19 M	19 M	23 F	25 M
Ancestry	Syrian Iraqi	CE	CE	Egypt	CE, African	CE	CE	CE	Asian	CE	CE, Asian	CE, Asian	CE	CE
Mutation Status	De novo	De novo	De novo	De novo	ND	De novo	De novo	ND	ND	De novo	De novo	De novo	De novo	De novo
Perinatal														
IUGR	−	−	−	−	−	+	−	+	U	+	−	−	−	−
Prematurity	−	−	−	+	−	−	+	+	U	−	−	−	−	−
Fetal Distress	+	−	+	+	+	+	+	+	U	+	−	−	+	+
Birth Wt (kg) (%ile)	2.9 (22)	3.24 (45)	3.865 (93)	2.235 (54)	3.288 (49)	2.759 (9)	3.32 (75)	2.28 (21)	U	2.52 (27)	2.824 (90)	2.7 (90)	2.95 (20)	4.25 (100)
Respiratory Distress	+	+	+	+	+	+	+	+	+	+	+	+	+	+
Tracheostomy	+	+	+	−	+	−	+	+	−	+	−	−	+	−
Age Trach removed					Capage 5			4		10			8	
NG or G tube	+	+	+	+	+	+	+	−	+	−	−	−	+	+
Facial dysmor	+	+	+	+	+	+	+	+	+	+	+	+	+	+
Growth (Z-scores for age at measurement)														
Height	−1.65	0.85	2.96	−0.52	−4.42	−1.5	−1.55	0.42	−5.69	−2.99	−1.52	−2.75	−0.47	−2.3
Weight	−2.05	−0.34	0.39	−1.08	−2.08	−1.5	−1.76	−0.48	−7.76	−3.04	−2.81	−1.33	−2.32	−1.41
BMI	−1.4	−1.06	−1.74	−2.05	1.84		−1.05	0.69	−1.59	−5.06	−3.48	0.4	−2.38	−0.26
Neurological														
Microcephaly	+	−	−	+	−	+	+	−	+	+	−	−	+	−
ID	TY	TY	TY	TY	+	+	+	+	+++	−	++	+++	+	++
Seizures	−	−	+	−	−	−	−	−	+	+	−	−	−	−
Handedness	TY	TY	TY	L	L	L	L	L	L	L	L	L	L	Poor use b/l
Cranial nerves														
I: Anosmia	TY	TY	TY	TY	−	−	+	−	+	+	+	+	+	−
III: CFEOM	+	+	+	+	+	+	+	+	+	+	+	+	+	+
Manual lid elevation	TY	+	U	+	−	+	−	+	U	+	−	−	+	+
VII: facial weakness	+	+	+	+	+	+	+	+	+	+	+	+	+	+
VIII: hearing loss	−	−	−	−	−	−	+	−	−	−	−	−	+	−
X: vocal cord paralysis	+	+	+	+	+	+	+	+	U	+	−	−	+	−
XII: swallowing dysfunction	+	+	+	+	−	+	+	−	+	+	−	−	+	−
Neuromuscular														
Clin PN Age detected	−	−	−	+1	−	+8	+4	+1	+<12	+12	+7	+7	+<17	+<12
EMG/NC abnl	ND	ND	ND	+	ND	ND	+	+	+	+	+	+	+	ND
JC: Upper Ex	+	+	+	+	+	−	+	+	−	−	+	+	+	+
JC: Lower Ex	+	+	+	−	−	−	+	+	+	+	+	−	+	+
Endocrine														
Hypogon adotropic hypogonadism									+	+	−	+	+	+
Olf dysgenesis	+	U	hypo	+	U	U	+	−	+	+	+	+	+	−
Microphallus	−	+	N/A	+	U	N/A	−	+	N/A	N/A	−	+	N/A	+
Cryptorchidism	−	−	N/A	−	+	N/A	−	+	N/A	N/A	−	+	N/A	+
Bone age	U	U	U	U	U	U	U	U	U	7.5 at 12.2	12.5 at 14.6	12.5 at 14.6	U	U
Osteopenia	U	−	−	U	U	U	U	U	+	+	+	+	+	U
GI														
Cyclic vomiting	−	−	−	−	−	−	−	+	+	+	−	−	−	−
Cardiac														
Pacemaker	−	−	−	−	−	−	−	−	−	−	−	−	−	+
Sinustachycardia	+	−	+	−	+	+	+	+	−	+	+	+	+	+
Ophthalmology														
Pupils	A	miotic	R	Min R, A	U	U	R	Poor dilation	U	Min R, A	R	R	Min R, A	R
BCVA (OD, OS)	Too young	Too young	20/130, 20/960	NFA	U	U	20/60 20/120	NLP, 20/100	U	20/63 20/63	20/80 20/80	20/400 20/400	20/100 20/40	20/40 20/30
Ptosis	+	+	+	+	+	+	+	+	+	+	+	+	+	+
Ptosis Surgery	+	+	−	−	+	+	U	+	U	+	+	+	−	+
Ocular Alignment	XT	XT	XT	XT	XT	XT	XT	XT	XT	XT	XT	XT	XT	XT
Horizontal Movement	none	min	min	min	min	min	min	min	min	min	min	min	None OS Min OD	min
Subject	I	II	III	IV	V	VI	VII	VIII	IX	X	XI	XII	XIII	XIV
Vertical movement	none	min	min	none	min	min	none	min	none	none	min	min	none	min
Strabismus Surgery	−	−	−	−	+	−	+	+	+	+	+	+	−	+
Corneal exposure	+		−	+	+		+	+	+	+	+	−	−	+
Other	G, En, atrophiciris, no foveal reflex	ONH		En, absent corneal sensation, large ON				Cataract	Ec	En, Ab	Small ON		Cataract	

**Table 2:** Neurocognitive, neurological and motor function of subjects evaluated at NIH

Subject	VII	X	XI	XII	XIII	
Age at Testing	7	12	14	14	19	
Cognitive Testing						
IQ test administered	WPPSI-IV	WISC-V	WISC-V	Mullen	WAIS-IV	
Full Scale IQ	65	96	41	17	73	
Verbal	73	103	45	18	83	
Nonverbal	68	88	44	15	86	
Adaptive Behavior Composite Score	79	76	50	37	62	
Emotional/behavioral concerns						
Internalization	41	66	52	71	59	
Externalization	44	46	54	69	50	
Attention deficit/hyperactivity	61	68	61	81	67	
Somatic	50	65	60	64	51	
Neuro exam						
Strength	full	Weak hip girdle		Reduced bulk	Weak hip girdle, ankles	
Reflexes	normal		Low ankle	brisk	increased	
Babinski	flexor	R upgoing		Bilateral up	Bilateral up	
Ankle clonus		1-2 beats	bilateral	2-3 beats	bilateral	
Sensation	decreased				decreased	
Motor Function						
Ambulation - independent	2-3’	1 mile	30m	crawls	25-30’	
Uses assistive device	Rollator	no	rollator	rollator	no	
Joint range of motion	Hamstring tightness	UL hypermobile Hamstring and Achilles tightness	Hamstring and Achilles tightness	UL hypermobile Hamstring and Achilles tightness	Hamstring and Achilles tightness	
Alignment	Genuvalgus, varus tibial bowing, LLD	LLD	LLD	“Windswept” hips, no LLD	genuvalgus	
						
Peripheral Nerve Conduction Studies						Normal values
Sensory						
Median Amp (μV) CV (m/s)	2 30	NR NR	NR NR	5 34	NR NR	≥ 15 ≥ 50
Sural Amp (μV) CV (m/s)	0.4 30	NR NR	2 24	NR NR	NR NR	≥ 6 ≥ 40
Motor						
Median Amp (μV) CV (m/s)	5.8 34	3.9 31	1.1 31	7 31	1.9 22	≥ 4.5 ≥ 50
Peroneal Amp (μV) CV (m/s)	1 29	NR NR	NR NR	NR NR	NR NR	≥ 2.5 ≥ 40
Facial						
R O. Oculi Amp (mV) Latency (ms)	ND	ND	0.7 1	1.2 1.2	0.2 3.3	≥ 1.0 ≥ 3.1
R O. Oris Amp (mV) Latency (ms)	ND	ND	0.7 1.1	0.5 1.1	0.9 2.1	≥ 1.0 ≥ 3.1
L O. Oculi Amp (mV) Latency (ms)	ND	ND	1.2 1.7	1.1 1.5	0.2 1.6	≥ 1.0 ≥ 3.1
L O. Oris Amp (mV) Latency (ms)	ND	ND	1.1 1.8	0.8 1.7	0.8 1.7	≥ 1.0 ≥ 3.1
Needle EMG						
				Tibialis anterior WNL	Vastus lateralis and tibialis anterior neurogenic;Biceps brachii WNL	

Note: Standard Scores on cognitive testing have a mean of 100, Standard Deviation of 15; WAIS-IV nonverbal IQ=Perceptual Reasoning; WPPSI-IV and WISC-IV nonverbal IQ=nonverbal index; Verbal=verbal comprehension. For emotional/behavioral concerns the Child Behavior Checklist was used. R – right L – left O. Oculi- Orbicularis oculi, O. Oris-Orbicularis OrisAmp-Amplitude, mV-millivolt, m/s-meters/second, NR – nonrecordable, ND – not done, LLD - leg length discrepancy, WNL - within normal limits

**Table 3:** MRI findings. NB: newborn; EOMs: extraocular muscles

Subject	I	IV	VI	VII	IX	X	XI	XII	XIII
Age at imaging	NB	12m	2y	7y	12y	12y	14y	14y	17y
Corpus callosum hypoplasia	+	+	+	+	+	+	+	+	+
Basal ganglia dysgenesis	+	+	+	+	+	+	+	+	+
Lateral ventricle asymmetry	+	+	+	+	+	+	+	+	+
Sylvian fissure asymmetry	+	+	+	+					
Hippocampal malrotation		+	+		+	−	−	+	+
White matter hypoplasia		+	+	+	+	+			
Absent olfactory bulbs and sulci	+	+		+	+	+	+	+	+
Cerebellar asymmetry/dysmorphism	+	+	−	+	+	+	+	+	+
Pons small	+	+	+	+			−	−	+
CN3 absent		+		+	+	+	+	+	+
CN7 absent/hypoplastic		+		+	+	+	+	+	+
CN8 abnormal				+		−			+
Cochlea abnormal				+					+
EOMs small	+	+	+	+	+	+	+	+	+

**Table 4:** Distinguishing characteristics between TUBB3 R262H syndrome and other syndromes in the differential diagnosis. AD: autosomal dominant, AR: autosomal recessive, + finding present, − finding not reported, +/− present in some individuals.

	TUBB3 R262H	TUBB3 E410K	TUBB3 R262C	Classic Moebius	Carey-Fineman-Ziter syndrome	Arthrogryposis, distal, 5D	Marden Walker
Phenotype MIM #	600638	600638	600638	157900	254940	605896	248700
Gene	*TUBB3*	*TUBB3*	*TUBB3*	N/A	*MYMK*	*ECEL1*	*PIEZO2*
Inheritance	De novo	De novo	AD	sporadic	AR	AR	sporadic
Ptosis	+	+	+/−	−	+/−	+	+
Limited Horizontal Eye Movements	+	+	+/−	+	+/−	+/−	−
Limited Vertical Eye Movements	+	+	+	−	−	+/−	−
Facial Weakness	+	+	−	+	+	−	+
Facial dysmorphisms	+	+	−	+/−	+	−	+
Micrognathia	−	−	−	+	+	−	+
Robin sequence	−	−	−	+/−	+	−	−
Intellectual Disability	+	+	−	+/−	−	−	+
Congenital Contractures	+	−	−	+/−	+	+	+
Progressive peripheral axonal neuropathy	+	+	−	−	−	−	−
Myopathy	−	−	−	−	+	−	−
Cryptorchidism	+	+	−	−	+	−	−
Hypogonadotropic hypogonadism	+	+	−	−	−	−	−
Brain MRI Findings							
Corpus callosum hypoplasia	+	+	+/−	−	−	−	+
Basal Ganglia dysgenesis	+	−	−	−	−	−	−
Dandy Walker malformation	−	−	−	−	−	−	+/−

## Data Availability

The data are not publicly available to protect subject privacy.
